# Case Report: A Missense Mutation in Dyskeratosis Congenita 1 Leads to a Benign Form of Dyskeratosis Congenita Syndrome With the Mucocutaneous Triad

**DOI:** 10.3389/fped.2022.834268

**Published:** 2022-04-06

**Authors:** Liqing Wang, Jianwei Li, Qiuhong Xiong, Yong-An Zhou, Ping Li, Changxin Wu

**Affiliations:** ^1^The Key Laboratory of Chemical Biology and Molecular Engineering of Ministry of Education of China, Shanxi University, Taiyuan, China; ^2^The Key Laboratory of Medical Molecular Cell Biology of Shanxi Province, Institute of Biomedical Sciences, Shanxi University, Taiyuan, China; ^3^Bluttransfusion, The Second Hospital, Shanxi Medical University, Taiyuan, China

**Keywords:** dyskeratosis congenita syndrome, *DKC1*, missense mutation, c.1156G > A, p.Ala386Thr

## Abstract

**Background:**

Dyskeratosis congenita (DC) is a rare inheritable disorder characterized by bone marrow failure and mucocutaneous triad (reticular skin pigmentation, nail dystrophy, and oral leukoplakia). Dyskeratosis congenita 1 (*DKC1*) is responsible for 4.6% of the DC with an X-linked inheritance pattern. Almost 70 *DKC1* variations causing DC have been reported in the Human Gene Mutation Database.

**Results:**

Here we described a 14-year-old boy in a Chinese family with a phenotype of abnormal skin pigmentation on the neck, oral leukoplakia, and nail dysplasia in his hands and feet. Genetic analysis and sequencing revealed hemizygosity for a recurrent missense mutation c.1156G > A (p.Ala386Thr) in *DKC1* gene. The heterozygous mutation (c.1156G > A) from his mother and wild-type sequence from his father were obtained in the same site of *DKC1*. This mutation was determined as disease causing based on *silico* software, but the pathological phenotypes of the proband were milder than previously reported at this position (HGMDCM060959). Homology modeling revealed that the altered amino acid was located near the PUA domain, which might affect the affinity for RNA binding.

**Conclusion:**

This *DKC1* mutation (c.1156G > A, p.Ala386Thr) was first reported in a Chinese family with mucocutaneous triad phenotype. Our study reveals the pathogenesis of *DKC1* c.1156G > A mutation to DC with a benign phenotype, which expands the disease variation database, the understanding of genotype–phenotype correlations, and facilitates the clinical diagnosis of DC in China.

## Introduction

Dyskeratosis congenita (DC) is a rare inheritable disorder characterized by bone marrow failure and mucocutaneous triad (skin pigmentation, dystrophy nails, oral leukoplakia) (1). So far, several genes have been identified to be associated with DC, including dyskeratosis congenita 1 (*DKC1*), CTS telomere maintenance complex component 1 (*CTC1)*, regulator of telomere elongation helicase 1 (*RTEL1*), TERF 1-interacting nuclear factor 2 (*TINF2*), telomerase RNA component (*TERC*), telomerase reverse transcriptase (*TERT*), adrenocortical dysplasia homolog (*ACD*), NHP2 ribonucleoprotein (*NHP2*), NOP 10 ribonucleoprotein (*NOP10*), poly(A)-specific ribonuclease (*PARN*), nuclear assembly factor 1 (*NAF1*), and WD repeat containing antisense to TP53 (*TCAB1*), and DKC1 is responsible for 4.6% of the DC (2, 3). Almost 70 dyskeratosis congenita 1 (DKC1) variations causing DC have been reported in the Human Gene Mutation Database (HGMD^1^); the gene encoding a nucleolar protein is called dyskerin, which is involved in both ribosome biogenesis (4) and telomere maintenance (5). Here, we found a DC patient in a Chinese family. The clinical data of the patient and literature review of DC are described.

## Case Presentation

### Clinical Manifestations and Family History

**FIGURE 1:**
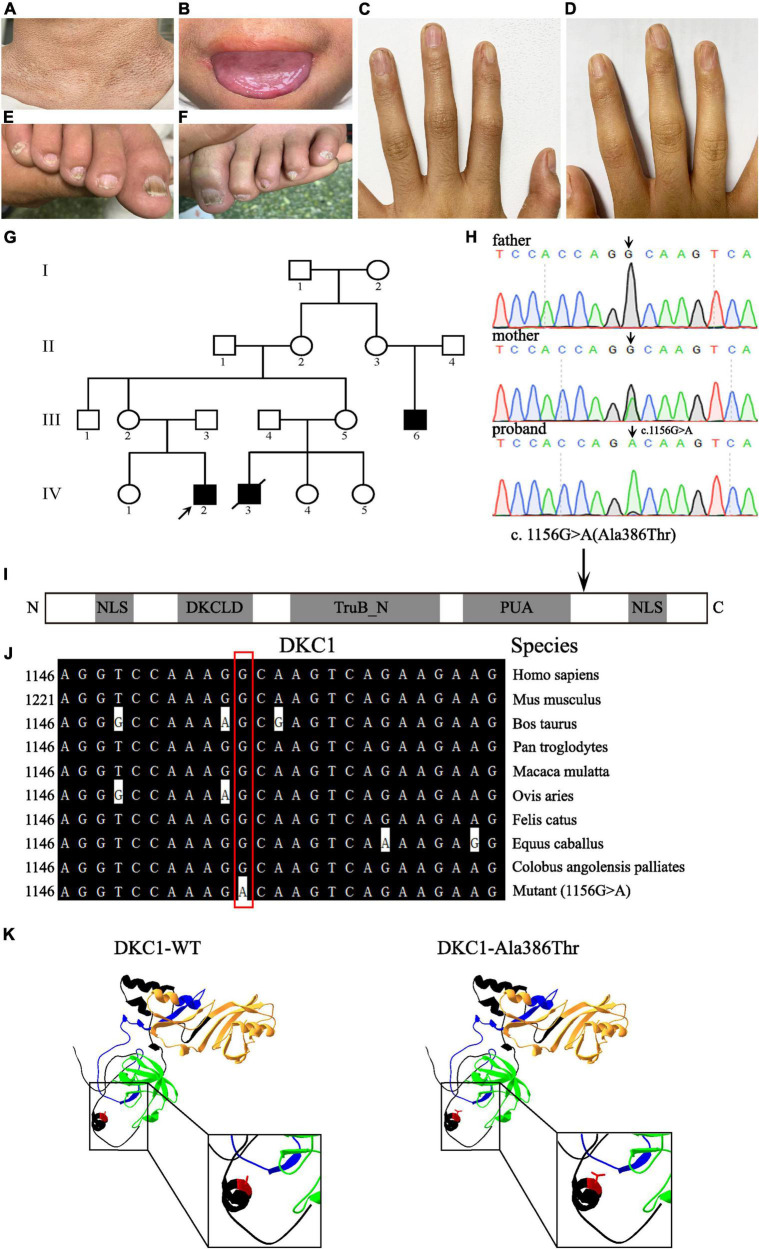
Clinical features of the proband and pedigree, sequencing analysis, and DKC1 mutation investigations. Pigmentation on the neck **(A)**, mucosal leukoplakia on the tongue **(B)**, finger nail ridging, toenail ridging, and longitudinal splitting **(C–F)** in the proband. **(G)** The pedigree of the family. The arrow indicates the proband. **(H)** Sequencing chromatograms show the proband with a hemizygous mutation DKC1 c.1156G > A, the proband’s mother with the same heterozygous mutation; the black arrow indicates the position of the nucleotide mutation. **(I)** A linear representation of the DKC1 protein shows the location of the N-terminal nuclear localization signals (NLS), DKCLD, TruB_N, and PUA domains. The black arrow shows the positions of the amino acid substitutions. **(J)** The mutant site (c.1156G > A) of DKC1 is highly conserved phylogenetically among the indicated species. **(K)** The mutant proteins were structured by the Swiss-Model online software and compared with the wild type. Ribbon representation of the human DKC1 and map of the studied variant localization obtained by homology modeling analysis. The wild-type and mutant monomers are shown in black; DKCLD, TruB_N, and PUA domains are shown in blue, orange, and green, respectively. Amino acid Ala386 is shown as red.

Three affected males (III-6, IV-2, and IV-3) and 14 unaffected individuals are involved in this family and are recruited from Shanxi Province, China (Figure 1G). The proband IV-2 is a 14-year-old boy with abnormal skin pigmentation on the neck (Figure 1A), oral leukoplakia (Figure 1B), and nail dysplasia on his hands and feet (Figures 1C–F). III-6 presents with similar phenotypes. II-2, II-3, III-2, and III-5 are mutation carriers without any mild signs of congenital dyskeratosis.

### Sequencing Analysis of the Patient and His Family

Whole-exome sequencing (WES) data were functionally annotated and filtered using cloud-based rare disease NGS analysis platform,^2^ based on the Ensembl (GRCh37/hg19), dbSNP, EVS, 1000 genome, ExAC, and GnomAD databases. Exonic sequence alterations and intronic variants at exon–intron boundaries, with unknown frequency or minor allele frequency (MAF) < 1% and not present in the homozygous state in those databases, were retained. Filtering was performed for variants in genes associated with DC. Then the only DC-related gene mutation *DKC1* mutation (c.1156G > A, p.Ala386Thr) was identified.

Peripheral blood samples were collected from this family, which includes three individuals (III-2, III-3, and IV-2); a recurrent *DKC1* hemizygous mutation (c.1156G > A) in exon 12 was confirmed in the proband (IV-2) by using Sanger sequencing (Figure 1H). Furthermore, a heterozygous mutation (c.1156G > A) in his mother (III-2) and a wild-type sequence in his father (III-3) were obtained on the same site of *DKC1* (Figure 1H). The original contributions presented in the study are publicly available. These data can be found here: ClinVar Wizard Submission ID: SUB11097305; Accession: SCV002097631.

### Pathogenicity Prediction of Variant

The effect of the missense variant was computationally analyzed by four prediction programs: Mutation Taster, SIFT, PolyPhen-2, and PROVEAN. The outcomes are summarized in Table 1.

**TABLE 1:** Bioinformatics prediction of a pathogenic variant.

Mutation prediction	Prediction score values			
Tool	Mutation Taster	SIFT	PolyPhen-2	PROVEAN
c.1156G > A	D (0.99)	D (0.02)	B (0.122)	D (−3.121)

*Mutation Taster: D, disease causing; P, polymorphism.*

*SIFT: D, damaging; T, tolerated.*

*PolyPhen-2: D, probably damaging; P, possibly damaging; B, benign.*

*PROVEAN: D, deleterious; N, neutral.*

### Molecular Analysis

Evolutionary conservation of amino acid residue showed that the impaired amino acid residues Ala386 were highly conserved in different species (Figure 1J). The eukaryotic DKC1 protein presents three well-characterized domains: DKCLD (amino acids 49–106), TruB_N (amino acids 107–247), and PUA (amino acids 297–371) besides nuclear and nucleolar localization signals (amino acids 11–20; 446–458) (6, 7). Bioinformatic and biochemical assessment on the effect of the altered amino acid on the functions of DKC1 shows that the missense mutation was concentrated near the PUA domain (Figures 1I,K), which is crucial for the RNA binding of telomerase (7). *DKC1* mutations concentrated in or near the PUA domain decrease the affinity for RNA binding (6). In conclusion, the recurrent *DKC1* pathogenic variant was identified by WES and Sanger sequencing in a Chinese DC family.

## Discussion

Here, we report a case of DC in a Chinese pedigree with a mutation c.1156G > A (p.Ala386Thr) in *DKC1*. The affected amino acids are located near the PUA kinase domain from the linear structure, indicating that the mutation might result in defect on the affinity for RNA binding (6). Evolutionary conservation analysis of amino acid residue showed that the amino acid residue Ala386 is highly conserved among DKC1 protein from different species, indicating that the mutation is likely pathological.

We have reviewed articles describing cases of DC using the Human Gene Mutation Database and NCBI—PubMed, with the search term “dyskeratosis congenita” from January 1998 to November 2021 (Table 2). Among the studies, we identified 74 variations in *DKC1* with 85 individuals for analysis. Most publications were case reports so that the clinical data were not comprehensive. There were 87.5% male patients, 12.5% female patients, and 29 patients without gender description in the patients, indicating that males were the dominant patients of DC.

**TABLE 2:** Main clinical features of dyskeratosis congenita (DC) patients in the Human Gene Mutation Database (HGMD)/literature.

Mutation on cDNA/protein	Ethnic origin	Gender	Age (years)	Mucocutaneous triad	Bone marrow failure	Anemia	Thrombocytopenia	Telomere shortening	Pulmonary fibrosis	References
5C > T/A2V	Egyptian	Male	40	+	NT	NT	NT	NT	+	(8)
29C > T/P10L	-	-	-	+	+	NT	NT	NT	NT	(4)
91C > A/Q31K	Japan	Male	11	+	NT	NT	+	NT	NT	(9)
91C > G/Q31E	United States	Male	33	+	+	NT	NT	+	NT	(10)
106T > G/F36Y	Belgian	Male	30	+	NT	NT	+	NT	+	(11)
113T > C/I38T	Italy	Male	0.75	+	+	+	NT	NT	NT	(11)
114C > G/I38M	United Kingdom	-	-	+	+	NT	NT	NT	NT	(12)
115A > G/K39E	United Kingdom	Male	-	-	-	-	-	-	-	(13)
119C > G/P40R	United Kingdom	Male	14	+	NT	NT	NT	-	-	(14)
121G > A/E41K	Turkey	Male	-	-	-	-	-	-	-	(13)
127A > G/K43E	Germany	-	-	-	-	-	-	-	-	(15)
146C > T/T49M	United Kingdom	Male	3	NT	NT	NT	NT	NT	NT	(16)
			2.6	NT	NT	NT	NT	NT	NT	(16)
			5	+	NT	NT	NT	NT	NT	(16)
145A > T/T49S	United States	Male	49	NT	+	+	+	+	+	(1)
		Female	25	NT	NT	NT	NT	NT	NT	(1)
160C > G/L54V	United States	Female	65	+	NT	+	NT	NT	NT	(17)
		Female	65	+	NT	NT	NT	NT	NT	(17)
		Male	45	+	+	NT	NT	+	NT	(17)
166_167invCT/L56S	Russian	Male	14	+	NT	+	+	NT	NT	(18)
189T > G/N63K	Canada	Male	24	+	+	+	+	+	+	(19)
194G > A/R65K	Japan	Male	46	+	+	NT	NT	+	+	(20)
194G > C/R65T	Germany	-	-	-	-	-	-	-	-	(13)
198A > G/T66A	United States	-	-	-	-	-	-	-	-	(13)
200C > T/T67I	-	-	-	+	+	NT	NT	NT	NT	(4)
204C > A/H68Q	-	-	-	+	+	NT	NT	NT	NT	(4)
203A > G/H68R	Spain	Male	36	+	NT	+	+	NT	NT	(21)
202C > T/H68Y	United States	Male	-	NT	+	NT	NT	NT	NT	(12)
209C > T/T70I	United States	-	-	-	-	-	-	-	-	(22)
214C > T/L72F	China	Male	7	+	+	+	+	NT	NT	(23)
227C > T/S76L	United States	-	-	NT	+	NT	NT	NT	NT	(12)
361A > G/S121G	United Kingdom	Male	1.5	NT	NT	NT	NT	NT	NT	(16)
247C > T/R158W	United States	-	-	-	-	-	-	-	-	(24)
838A > C/S280R	United States	-	-	-	-	-	-	-	-	(24)
911G > A/S304N	United States	Male	-	-	-	-	-	-	-	(25)
941A > G/K314R	-	-	-	+	+	NT	NT	NT	NT	(4)
942G > A/K314K	United States	Male	65	NT	+	NT	+	+	+	(26)
949C > G/L317V	United States	Male	-	-	-	-	-	-	-	(25)
949C > T/L317F	Germany	-	-	-	-	-	-	-	-	(27)
961C > A/L321I	China	Male	4.3	+	NT	+	NT	NT	NT	(28)
961C > G/L321V	Italy	Male	-	-	-	-	-	-	-	(13)
965G > A/R322Q	Germany	-	-	-	-	-	-	-	-	(27)
1049T > C/M350T	United Kingdom	-	-	-	-	-	-	-	-	(13)
1050G > A/M350I	Austria	-	-	-	-	-	-	-	-	(13)
1050G > C/M350I	Germany	Male	40	+	NT	+	+	NT	NT	(29)
1051A > G/T351A	-	Male	7	+	NT	+	NT	NT	NT	(30)
1054A > G/T352A	-	Male	31	+	NT	NT	NT	NT	NT	(3)
1058C > T/A353V	Brazil	Male	3	+	+	+	NT	NT	+	(31)
	India	Male	12	+	NT	NT	NT	NT	NT	(32)
1066T > C/S356P	Portugal	Male	15	+	NT	NT	+	NT	NT	(33)
			10	+	+	NT	+	NT	NT	(33)
1069A > G/T357A	Japan	Male	10	+	+	+	+	NT	NT	(9)
1072T > G/C358G	Germany	Male	0.6	+	NT	NT	+	NT	NT	(34)
1075G > A/D359N	-	-	-	+	NT	NT	NT	NT	NT	(4)
1133G > A/R378Q	United States	-	-	NT	+	NT	NT	+	NT	(12)
1151C > T/P384L	United States	-	-	-	-	-	-	-	-	(24)
1156G > A/A386T	-	-	-	+	NT	NT	NT	NT	NT	(4)
1186G > A/K390Q	United States	-	-	-	-	-	-	-	-	(22)
1177A > T/I393F	India	Male	21	+	NT	+	NT	+	NT	(35)
1193T > C/L398P	Japan	Male	-	-	-	-	-	-	-	(36)
1204G > A/G402R	India	-	-	-	-	-	-	-	-	(13)
1205G > A/G402E	United States	Male	-	-	-	-	-	-	-	(37)
1213A > G/T405A	United States	Male	65	+	NT	NT	NT	NT	+	(38)
			69	NT	NT	NT	NT	NT	+	(38)
1223C > T/T408I	-	-	-	+	NT	NT	NT	NT	NT	(4)
1226C > G/P409R	United States	Male	46	+	NT	NT	NT	NT	NT	(1)
		Male	40	+	NT	NT	NT	NT	NT	(1)
		Female	16	+	NT	+	NT	NT	NT	(1)
		Female	16	+	NT	NT	NT	NT	NT	(1)
		Female	8	+	NT	NT	NT	NT	NT	(1)
	China	Male	24	+	NT	NT	NT	NT	NT	(7)
			20	+	NT	NT	+	NT	NT	(7)
1226C > TP409L	China	Male	20	+	NT	NT	NT	NT	NT	(39)
IVS1 ds592C_G	Belgium	Male	30	+	NT	NT	+	NT	+	(24)
			10	NT	NT	NT	+	NT	NT	(24)
			56	+	NT	NT	NT	NT	+	(24)
IVS2 as-15 T-C	China	Male	8	+	+	NT	NT	NT	NT	(40)
IVS2 as-5 C-G	Spain	-	-	-	-	-	-	-	-	(13)
IVS12 ds + 1 G-A/A386fsX1	Italy	Male	0.3	NT	NT	NT	NT	NT	NT	(41)
IVS14 as-2 A-G	-	-	-	+	+	NT	NT	NT	NT	(4)
-141C > G	Spanish	Male	13	NT	NT	NT	NT	+	NT	(24, 42)
-141C > G	United States	-	2	NT	NT	NT	NT	NT	+	(22)
103_105delGAA/E35del	United States	Female	10	+	NT	NT	NT	NT	NT	(17)
106_108delCTT/L36del	Caucasian	-	-	-	-	-	-	-	-	(37)
1168_1170delAAG/K390del	Spanish	Male	32	+	NT	NT	NT	NT	NT	(21)
1495_1497delAAG/K499del	United Kingdom	-	-	-	-	-	-	-	-	(43)
112_116delATCAAinsTCAAC/ T38SfsX31	Canada	Male	-	-	-	-	-	-	-	(44)
14_215CT > TA/L72Y	United Kingdom	Male	-	-	-	-	-	-	-	(37)
1258,1259AG > TA/S420Y	-	-	-	+	+	NT	NT	NT	NT	(4)
Duplication of ∼14 kb (described at genomic DNA level)	United States	Male	-	NT	NT	NT	NT	NT	NT	(45)
1493A > G/S485G	Germany	-	-	NT	NT	NT	NT	NT	NT	(46)

*+, presents positive expression; NT, presents negative expression; -, presents not in detail.*

We find that the clinical symptoms of these DC patients are varied, but skin pigmentation, nail dystrophy, mucosal leukoplakia, and bone marrow failure are the most classic symptoms in patients. In this analysis, the incidence of skin pigmentation, nail dystrophy, and mucosal leucoplakia are nearly 86.58, 78.048, and 64.63%, respectively. Moreover, apart from the mucocutaneous triad, anemia can be another routine clinical sign of DC. Missense mutation is the most common mutation type among all the variations and shows higher incidence of the typical clinical symptoms of DC, but only one patient with c.194G > C (p.R65K) had mild symptoms such as pulmonary symptoms (20). The patient with mutation of small indel (c.166_167invCT) only suffer from thrombocytopenia and anemia (18). The patients with mutations of regulatory (c.-142C > G or c.-141C > G) only suffer from short telomere or pulmonary fibrosis (22, 24).

We also found 13 variants of *DKC1* in Asia with 100% male (7, 9, 13, 20, 23, 28, 32, 35, 36, 40), 52 variants in non-Asia with 84.8% male (1, 8, 10–14, 16–19, 21, 24–26, 29, 31, 33, 34, 37, 38, 41, 42, 44, 45), and 10 variants with unknown nationality (3, 4, 30). Asians develop DC at a younger age than non-Asians, between 4.3 and 46 years old (1, 7–12, 14, 16–24, 26, 28, 29, 31–35, 38–42). The incidence of the mucocutaneous triad (skin pigmentation, nail dystrophy, and mucosal leukoplakia), bone marrow failure, thrombocytopenia, and telomere shortening in Asia are similar to that of non-Asia (Table 3; 1, 3, 4, 7–12, 14, 16–24, 26, 28–35, 38–42, 45, 46). However, the DC-Asians are more likely to develop anemia instead of pulmonary fibrosis than non-Asians apart from the mucocutaneous triad (Table 3; 1, 3, 4, 7–12, 14, 16–24, 26, 28–35, 38–42, 45, 46). Unfortunately, the patient involved in our study did not present with anemia; the reason could be due to the lower incidence (35.7%) of anemia in Asian DC population.

**TABLE 3:** The Asian and outside Asian variations and the main clinical phenotypes.

Mutation percent	Age (year)	Male	Mucocutaneous triad	Bone marrow failure	Anemia	Thrombocytopenia	Telomere shortening	Pulmonary fibrosis
Asian (19.07%)	4.3–46	100%	78.57%	35.7%	35.7%	28.57%	14.28%	7.17%
Outside Asian (81.11%)	0.3–69	84.09%	70.59%	37.5%	19.6%	27.45%	13.72%	23.53%

The *DKC1* variation of c.1156G > A (p.Ala386Thr) was also reported from a DCR216-family in 2006 (4). The patient presents both the features of classic DC and Hoyeraal Hreidarsson (HH) syndrome, including intrauterine growth retardation, developmental delay, microcephaly, cerebellar hypoplasia, immunodeficiency, or bone marrow failure (4). However, the patient involved in our study only presents with benign phenotype of the mucocutaneous triad without any other abnormality, which provides more information on the mutation phenotype spectrum of DC. A similar case occurs for the *DKC1* c.1226C > G (p.P409R) mutation. This mutation was first identified in the patient with the features of liver cirrhosis, frequent caries, low platelets, gray hair, and tongue cancer in 2013 (1). However, the patient with the same mutation was reported from China in 2020 presenting fewer symptoms of reticulate interspersed pigmentation with hypopigmented macules on the neck, fingernail ridging and longitudinal splitting, and mucosal leukoplakia on the tongue (7). Those results demonstrate that there is no specific relationship between the genotype and phenotype.

Our findings indicate *DKC1* missense mutation c.1156G > A leads to a benign phenotype, which expands the disease variation database, the understanding of genotype–phenotype correlations, and facilitates the clinical diagnosis of DC in China. However, the mechanism of *DKC1* mutation resulting in DC should be investigated further.

## Data Availability Statement

The datasets presented in this study can be found in online repositories. The names of the repository/repositories and accession number(s) can be found below: Clinvar [accession: SCV002097631].

## Ethics Statement

The studies involving human participants were reviewed and approved by the ethics committee of Shanxi University (SXULL2021080). Written informed consent to participate in this study was provided by the participants’ legal guardian/next of kin. Written informed consent was obtained from the minor(s)’ legal guardian/next of kin for the publication of any potentially identifiable images or data included in this article.

## Author Contributions

LW wrote the manuscript and performed the practical work. JL collected patients’ data. PL and QX analyzed the patients’ data. PL designed the study. Y-AZ, PL, and CW conceived the study and edited the manuscript. All authors contributed to the article and approved the submitted version.

## Conflict of Interest

The authors declare that the research was conducted in the absence of any commercial or financial relationships that could be construed as a potential conflict of interest.

## Publisher’s Note

All claims expressed in this article are solely those of the authors and do not necessarily represent those of their affiliated organizations, or those of the publisher, the editors and the reviewers. Any product that may be evaluated in this article, or claim that may be made by its manufacturer, is not guaranteed or endorsed by the publisher.

## Footnotes

1 http://www.hgmd.org/; 2021.2.

2
http://www.gene.ac/
